# Neoadjuvant combined immunotherapy with intratumoral TransCon TLR7/8 Agonist and systemic TransCon IL-2 β/y or pembrolizumab in patients with locally advanced oral cancer—a monocentric experience

**DOI:** 10.1007/s00262-026-04515-8

**Published:** 2026-09-04

**Authors:** Manuel Weber, Leah Trumet, Fabienne Lange, Julia Murk, Philipp Schubert, Hasan Alsamra, Joy Backhaus, Stefanie Corradini, Marco Kesting, Sarina Müller, Joan Morris, Marlen Haderlein

**Affiliations:** 1 Department of Oral and Cranio-Maxillofacial Surgery, Uniklinikum Erlangen, Friedrich-Alexander-Universität Erlangen-Nürnberg (FAU), 91054 Erlangen, Germany; 2 Deutsches Zentrum Immuntherapie (DZI) and Comprehensive Cancer Center Erlangen-EMN (CCC ER-EMN), Friedrich-Alexander-Universität Erlangen-Nürnberg (FAU), Erlangen, Germany; 3 Department of Operative Dentistry and Periodontology, Uniklinikum Erlangen, Friedrich-Alexander-Universität Erlangen-Nürnberg (FAU), Erlangen, Germany; 4 Institute of Pathology, Universitätsklinikum Erlangen, Friedrich-Alexander-Universität Erlangen-Nürnberg (FAU), Erlangen, Germany; 5 Department of Radiation Oncology, Uniklinikum Erlangen, Friedrich-Alexander-Universität Erlangen Nürnberg (FAU), Erlangen, Germany; 6 Institute of Medical Teaching and Medical Education Research, University Hospital of Würzburg, Würzburg, Germany; 7 Department of Otolaryngology, Head and Neck Surgery, Uniklinikum Erlangen, Friedrich-Alexander-Universität Erlangen-Nürnberg (FAU), Erlangen, Germany; 8 Former Employee of Ascendis Pharma, Palo Alto, CA USA

**Keywords:** Local immune activation, Oral cancer, HNSCC, OSCC

## Abstract

**Purpose:**

Combination immunotherapy has shown encouraging activity across multiple solid tumors. We report the complete and consecutive cohort of patients with locally advanced oral squamous cell carcinoma (OSCC) treated at a single center within the prospective, multicenter, randomized phase 2 BelieveIT-201 trial (ASND0038), evaluating neoadjuvant intratumoral Toll-like receptor (TLR) 7/8 agonist (TransCon TLR7/8 Agonist) therapy combined with systemic immunotherapy.

**Methods:**

Patients with non-metastatic OSCC treated within the BelieveIT-201 trial (ASND0038) between April and December 2024 were included. Neoadjuvant therapy comprised two cycles of intratumoral TransCon TLR7/8 Agonist combined with either intravenous pembrolizumab or TransCon IL-2 β/γ according to 1:1 randomization, followed by surgical resection. The trial was terminated prematurely by the sponsor for reasons unrelated to safety or efficacy, which limited the cohort to six patients. Clinical, radiographic, pathological responses, and safety were assessed. Immunohistochemical analyses of paired pre- and post-treatment tumor samples evaluated immune cell infiltration (CD3, CD8, CD68, CD163). The individual patient was the unit of analysis, and given the small number of patients all analyses are descriptive; no inferential statistical testing was performed. Progression-free survival (PFS) and overall survival (OS) are reported as absolute event counts.

**Results:**

Six patients were treated (median age 63 years; median follow-up 81 weeks). Three patients achieved a major clinical response, two showed partial response, and one had progressive disease. Pathologic evaluation revealed one complete response, one major response, and four non-responses. All patients underwent surgery; postoperative morbidity was substantial, with at least one grade III adverse event in every patient and one postoperative death. All three patients with a major clinical response developed sterile tumor-associated pseudoabscesses in spatial proximity to the injection site. Immunohistochemical analyses revealed remodeling of the tumor immune microenvironment, including increased T-cell infiltration, most pronounced in the tumor center. Within the first year, two of six patients experienced a progression-free survival event (*n* = 1 progressive disease, *n* = 1 death). After surgery none of the patients showed disease recurrence.

**Conclusion:**

Neoadjuvant intratumoral TransCon TLR7/8 Agonist-based combination immunotherapy was feasible in this small prospective cohort of patients with locally advanced OSCC and was accompanied by consistent remodeling of the tumor immune microenvironment. Because of the limited number of patients and the premature termination of the parent trial, no conclusions on efficacy or on comparative tolerability can be drawn.

## Introduction

Standard of care for locally advanced oral squamous cell carcinoma (OSCC) is surgery followed by radio- or radiochemotherapy. Despite continuous advances in the individual therapeutic modalities, patients with OSCC exhibit relatively high 2-year locoregional recurrence rates up to 30% after surgery and radio(chemo)therapy [1].

In metastatic head and neck squamous cell carcinoma (HNSCC) including OSCC, immunotherapy with the PD1-inhibitors pembrolizumab and nivolumab is approved and established in palliative standard treatment [2, 3]. In contrast, in the curative setting, studies evaluating the addition of checkpoint inhibitors to cisplatin-based definitive radiochemotherapy, followed by maintenance immunotherapy, failed to meet their primary endpoints [4–6].

The primary aim of most current neoadjuvant immunotherapy protocols in HNSCC is not merely to achieve tumor shrinkage or improve operability with less extensive surgery, but rather to establish durable tumor control and ultimately improve survival. The KEYNOTE-689 trial [7], which enrolled approximately 60% of patients with OSCC, met its primary endpoint by demonstrating an event-free survival benefit for perioperative pembrolizumab (neoadjuvant plus adjuvant) followed by surgery compared with surgery alone. As a result, perioperative pembrolizumab has become the standard of care for patients with locally advanced (UICC stage III–IV), resectable OSCC with PD-L1 expression ≥ 1% (CPS). However, pathologic response rates to neoadjuvant immunotherapy in this study were modest: after two cycles of pembrolizumab, the major pathologic response rate was 9.3%, and the pathologic complete response rate was 3%. Notably, the observed survival benefit was largely driven by a reduction in distant metastases on longer-term follow-up [7].

Evidence from other solid tumors, such as melanoma, demonstrates that combination immunotherapy yields superior survival outcomes compared with monotherapy [8]. The phase II, BelieveIT-201 trial (ASND0038) evaluated a neoadjuvant combination immunotherapy using an intratumorally administered TransCon TLR7/8 Agonist in combination with either systemic pembrolizumab or a modified Interleukin-2 (IL-2) [TransCon IL-2 β/γ]. Early clinical data have demonstrated an antitumor response in patients with HNSCC undergoing immunotherapy with the TLR agonist plus IL-2 [9], as well as with the TransCon TLR7/8 Agonist plus pembrolizumab [10]. Importantly, combined immunotherapy demonstrated an acceptable safety profile.

The mechanistic hypothesis underlying the combination investigated here is the following. The efficacy of PD-1 blockade depends on the presence of a pre-existing, functionally competent intratumoral T-cell infiltrate [11]. Antigen presentation is frequently impaired in oral squamous cell carcinoma [12], and the extent of pre-existing immune infiltration varies substantially between tumors [13], which may contribute to the low pathological response rates observed after neoadjuvant checkpoint inhibition alone [14, 15]. Agonism of Toll-like receptors 7 and 8 activates innate immunity within the tumor, promotes maturation of dendritic cells and antigen presentation, and can thereby generate a locally inflamed microenvironment [16]. Because systemic administration of TLR7/8 agonists is limited by cytokine-mediated toxicity, intratumoral delivery from a sustained-release hydrogel carrier is intended to maintain high local concentrations while minimizing systemic exposure. The systemic combination partner is then intended to sustain and amplify the locally primed T-cell response, either by relieving PD-1-mediated inhibition or, in the case of TransCon IL-2 β/γ, by preferentially expanding cytotoxic effector cells while avoiding IL-2Rα-mediated expansion of regulatory T cells. The combination therefore addresses local priming and systemic effector expansion simultaneously, with the objective of increasing pathological response and locoregional control.

In the present report, we describe the complete and consecutive cohort of six patients treated at our institution within this prospective randomized phase 2 trial, receiving neoadjuvant combination immunotherapy incorporating TransCon TLR7/8 Agonist in combination with TransCon IL-2 β/γ or pembrolizumab. Given the small number of patients resulting from the premature termination of the trial, the analysis is descriptive and hypothesis-generating and is not intended to assess the efficacy of the treatment.

## Material and methods

### Study design and setting

This report presents the complete and consecutive cohort of patients treated at our institution within the prospective, multicenter, randomized, open-label phase 2 trial BelieveIT-201 (ASND0038, NCT05980598) between April and December 2024. All patients received intratumoral TransCon TLR7/8 Agonist and were randomized to receive either intravenous pembrolizumab or intravenous TransCon IL-2 β/γ as systemic combination partner. Randomization was performed centrally by the sponsor; no site-level allocation took place. Eligibility criteria, treatment schedule, timing of surgery, response criteria, safety follow-up and the translational endpoints were predefined in the trial protocol. No patient treated at our site was excluded from the present analysis. The trial was terminated prematurely by the sponsor for reasons unrelated to safety or efficacy, and enrollment at our site was therefore limited to six patients. The present analysis was not part of the predefined statistical analysis plan of the parent trial; it is a descriptive, hypothesis-generating single-site analysis that is neither powered for nor intended to assess efficacy.

Eligible patients had histologically confirmed, non-metastatic squamous cell carcinoma of the oral cavity, oro-/hypopharynx, or larynx (HPV-positive stage III or HPV-negative stage III–IVA), with at least one lesion safely accessible for intratumoral TransCon TLR7/8 Agonist injection. Patients with active autoimmune disease were excluded. These were the eligibility criteria of the parent trial. All six patients treated at our site had primary tumors of the oral cavity; in two patients the tumor extended contiguously into the oropharynx. The present analysis therefore reports exclusively on patients with oral squamous cell carcinoma.

#### Medication

**Fig. 1:**
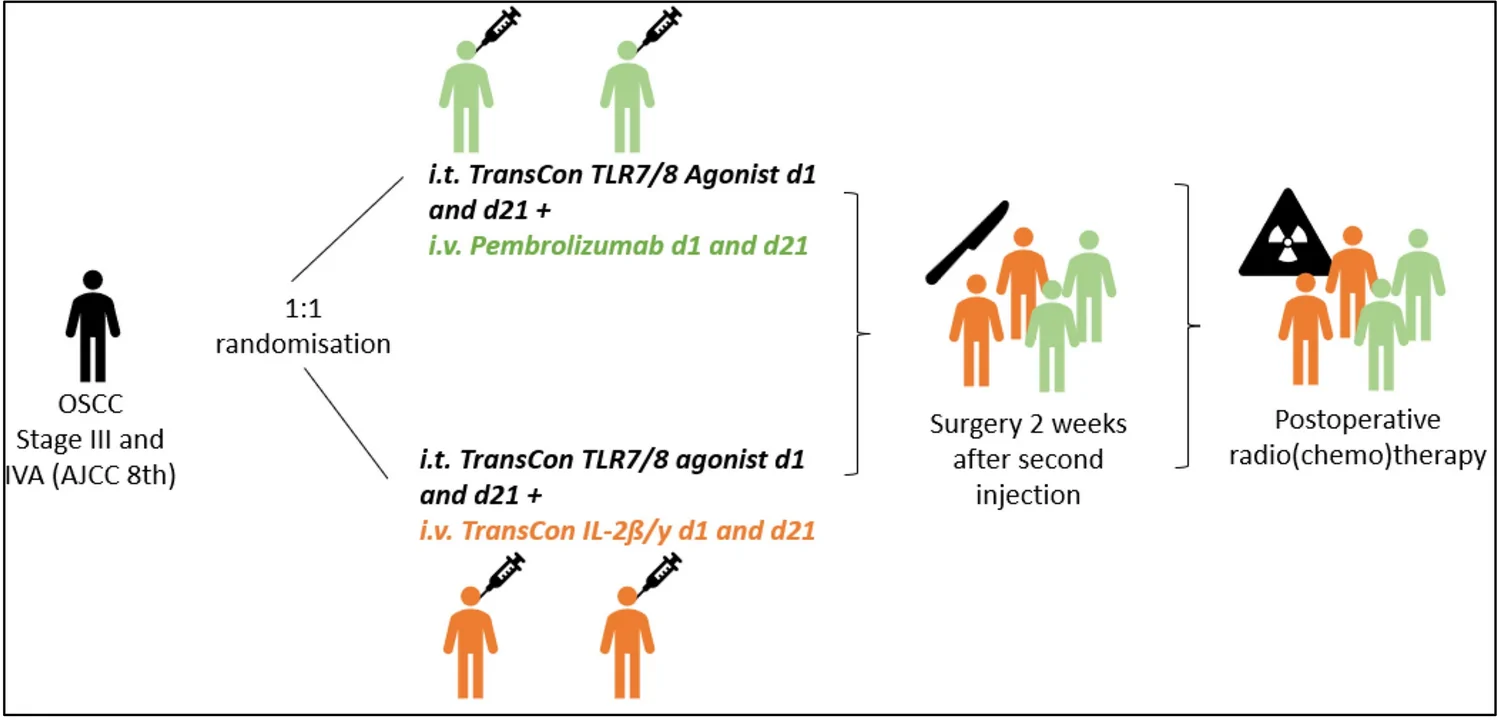
Treatment protocol, the figure shows the treatment protocol of our patient cohort receiving either intratumoral (i.t.) TransCon TLR7/8 Agonist and intravenous (i.v.) Pembrolizumab or intratumoral (i.t.) TransCon TLR7/8 Agonist and intravenous (i.v.) TransCon IL-2 β/y.

Two cycles of neoadjuvant immunotherapy were planned, consisting of TransCon TLR7/8 Agonist in combination with either TransCon IL-2 β/γ or pembrolizumab, according to randomization (see Fig. 1 for treatment overview). Each cycle was 21 days.

The TransCon TLR7/8 Agonist [16] is a Toll-like receptor 7/8 agonist composed of three components: resiquimod, a polyethylene glycol (PEG) hydrogel microparticle carrier, and a linker that is permanently bound to the carrier and transiently bound to resiquimod. This design enables sustained high local concentrations of resiquimod, potentially enhancing intratumoral immune activation while minimizing systemic exposure.

TransCon TLR7/8 Agonist was administered intratumorally on day 1 of each 21-day cycle at a dose of 0.5 mg in 1 mL per lesion. Up to five tumor lesions, including the primary tumor and involved lymph nodes, could be injected. Systemic therapy consisted of either pembrolizumab 200 mg intravenously or TransCon IL-2 β/γ 120 µg/kg intravenously on day 1 of each 21-day cycle.

TransCon IL-2 β/y [17] is a modified IL-2 in which binding to the IL-2 receptor α subunit is inhibited by introducing an engineered cysteine (where a 5 kDa mPEG is permanently attached) at position R38 within the IL-2Rα binding site. IL-2 β/γ is transiently conjugated to a branched 40kDa methoxy-poly (ethylene glycol) (mPEG) carrier, which shields receptor activation, modulates bioactivity, and prolongs systemic half-life. This design is intended to promote sustained antitumor activity while minimizing immunosuppressive effects and cytokine release associated with IL-2Rα engagement.

All patients received intratumoral TransCon TLR7/8 Agonist. In addition, patients were randomized 1:1 to receive either intravenous TransCon IL-2 β/γ or pembrolizumab 200 mg.

#### Surgery

Surgery was performed about 2 weeks after the second injection of TransCon TLR7/8 Agonist.

Pathologic response rates were defined as follows:

- Pathologic complete response: no vital tumor cells,
- Major pathologic response: ≤ 10% vital tumor cells,
- Pathologic partial response: > 10%, ≤ 50% vital tumor cells,
- Pathologic non-response: > 50% vital tumor cells

Resection specimens were examined according to the accredited routine protocol of the Institute of Pathology. The tumor bed was identified on haematoxylin–eosin sections by the presence of treatment-associated changes, including fibrosis, necrosis, histiocytic infiltration, and cholesterol clefts. Residual viable tumor was quantified as the percentage of the area of viable tumor cells relative to the total area of the tumor bed, assessed on all sections of the tumor bed. For the translational analyses, the invasive front was defined as the region within 100 µm of the invasive tumor margin, and the tumor center as the region of the tumor located at a distance from the invasive margin; both compartments were delineated on whole-slide scans by a pathologist prior to quantification.

Last safety follow-up was 28 days (± 7 days) after surgery. Afterward, in our department, patients have regularly follow-up visits every 6 weeks in the first 2 years and afterward every 6 months for up to 5 years.

#### Translational analyses

As translational part of the clinical study, various markers, including CD8, CD3, CD68, CD163, PD-1, and PD-L1 were immunohistochemically assessed in both the pre-immunotherapy biopsy specimens and the post-immunotherapy tumor resection samples in our center. CD3 and CD8 were used to identify total T lymphocytes and cytotoxic T cells, respectively. CD68 served as a marker for macrophages, while CD163 was employed to specifically detect M2-like, alternatively activated macrophages associated with immunoregulatory and tumor-promoting functions. Immunohistochemical staining was performed on 2 µm whole-slide sections using a Ventana Benchmark Ultra automated staining system (Ventana), in accordance with accredited staining protocols (https://www.dakks.de/en). The following primary antibodies were used: CD8 Dako M7103 1:100, CD3 abcam AB205228 1:5000, CD68 Dako PGM1 1:200, CD163 Novocastra 10D6 1:500, PD-1 CellMarque NAT105, PD-L1 Ventana SP263. Moreover, within the framework of the study, several markers and immune cell populations were centrally assessed in biopsy samples in the tumor epithelium, tumor stroma, and surrounding normal tissue prior to therapy, e.g., CD68, NKp46, CD303, CD3, FoxP3, Granzyme B, etc. These markers were correlated with the response to immunotherapy. Immunohistochemical analyses were performed on whole-slide sections. For each marker, a single labeling index (positive cells divided by all cells) was determined per patient and per tumor compartment (invasive front and tumor center) in the pre-treatment biopsy and in the post-treatment resection specimen. The unit of analysis was therefore the individual patient, and measurements from individual fields, regions of interest or cells were not treated as independent observations.

#### Statistics

Overall survival (OS), progression-free survival (PFS), and follow-up duration were measured from the date of first TransCon TLR7/8 Agonist injection to death, disease progression (including locoregional recurrence or distant metastasis), or last follow-up, whichever occurred first.

Given the small number of patients, all analyses are purely descriptive and no inferential statistical testing was performed. Continuous variables are reported as median and range, categorical variables as absolute numbers. Immunohistochemical results are reported as individual paired pre- and post-treatment labeling indices for each patient, separately for the invasive front and the tumor center, together with the median and range of the paired differences. Survival outcomes are reported as absolute event counts with the corresponding follow-up duration; Kaplan–Meier estimates are shown for illustration only, and no confidence intervals or comparative tests are reported, as these would not be meaningful in a cohort of six patients. No adjustment for multiple comparisons was applied, as no hypothesis tests were conducted. All findings are hypothesis-generating and require prospective validation.

## Results

Median follow-up is 22.5 months (range 2–27). This corresponds to 18.6 months; the shortest follow-up corresponds to the patient who died postoperatively.

6 patients with oral cancer were treated in our institution. 5 patients were male and 1 female. Median age was 63 years (range: 59 to 77years). For detailed patient information see Table 1.

**Table 1:** Table shows tumor localization and clinical stage, study medication, clinical/radiographic response, and pathologic response for each individual patient

Patient ID	Tumor localization and clinical stage, PD-L1 expression in biopsy	Study Medication	Clinical/radiographic response*	Pathologic response *, pathologic tumor stage, PD-L1 expression in primary tumor
1	Oral cavity Retromolar mandibular cT2cN2bcM0 G2 PD-L1 CPS 2	TransCon TLR7/8 Agonist and TransCon IL-2 β/y	Major response Pseudoabscess in CT scan preoperative	Major pathologic response ypT1ypN2b (3/125, ECE-) L0V0Pn0R0 PD-L1 CPS 25
2	Oral cavity anterior floor of mouth cT4cN1cM0 G3 PD-L1 CPS 1	TransCon TLR7/8 Agonist and Pembrolizumab	Progressive Disease	No response ypT3ypN3b(4/98 ECE +) L0V0Pn0 R0 PD-L1 CPS 25
3	Oral cavity Tongue and floor of mouth cT3cN2bcM0G2 PD-L1 CPS 6	TransCon TLR7/8 Agonist and TransCon IL-2 β/y	Major response Pseudoabscess in CT scan preoperative	No response ypT2ypN0(0/75) L0V0Pn0R0 PD-L1 CPS n.a
4	Oral cavity Planum buccale with infiltration of arcus palatoglossus cT1cN2bcM0 G2 PD-L1 CPS 20	TransCon TLR7/8 Agonist and Pembrolizumab	Major response Pseudoabscess in CT scan preoperative	Complete response ypT0ypN0 PD-L1 CPS 3
5	Oral cavity Tongue and arcus palatoglossus cT3cN2bcM0 G2 PD-L1 CPS 20	TransCon TLR7/8 Agonist and TransCon IL-2 β/y	Partial response	No response ypT2ypN2b(2/35)L0V0Pn0 R0 PD-L1 CPS 37
6	Oral cavity Arcus palatoglossus with oropharyngeal infiltration cT3cN1cM0 G2 PD-L1 CPS 27	TransCon TLR7/8 Agonist and Pembrolizumab	Partial response	No response ypT2ypN1(1/31)L0V0Pn1R0 PD-L1 CPS 100

^*^Radiographic and pathologic response is by local institutional evaluation

In all reported patients, the TransCon TLR7/8 Agonist was injected into the primary tumor but not into lymph node metastases.

Five patients received two cycles of neoadjuvant immunotherapy; one patient received only one cycle due to autoimmune hepatitis and was still requiring prednisolone at the time the second cycle would have been administered. All patients receiving TransCon IL-2 β/y experienced cytokine release syndrome (CRS) (grade 1: *n* = 2, grade 2: *n* = 1): One patient treated with pembrolizumab developed grade 1 CRS. Grade III toxicities after immunotherapy were reported in two patients. In one patient after first injection a seizure occurred (assumed to be related to opioid analgesic piritramid infusion after injection), and in the same patient after the second injection, a syncope and pseudoabscess were observed (both assumed to be related to injection of TransCon TLR7/8 Agonist). In another patient, a grade III urinary tract infection was diagnosed and was considered unrelated to immunotherapy.

Three of six patients demonstrated a major clinical response with clinical shrinkage of primary tumor and reduction in symptom burden, particularly pain. Two patients showed a partial clinical response, while one patient experienced progressive disease. All three patients with a major clinical response developed a tonsillar pseudoabscess in spatial proximity to the injection site (see Table 1), without any clinical signs of infection. Pseudoabscesses were observed in two of the three patients receiving TransCon IL-2 β/γ and in one of the three patients receiving pembrolizumab. No pseudoabscess was observed in the two patients with a partial response or in the patient with progressive disease.

All six patients underwent surgery. *N* = 6 patients required free flap reconstruction, *n* = 2 patients underwent mandibular box resection, and *n* = 1 patient mandibular box resection and partial maxillary resection. One patient died postoperatively due to cardiorespiratory decompensation, which was considered unrelated to immunotherapy. Among the surviving patients, the median duration of hospitalization was 18 days (12 to 35 days). Postoperatively, at least one grade III adverse event occurred in all patients: *n* = 4 wound healing complications, *n* = 1 bronchial infection, *n* = 1 arterial lung thromboembolism, *n* = 1 pneumonia, *n* = 1 sepsis, *n* = 1 acute kidney injury. All grade III° adverse events were considered to be unrelated to immunotherapy.

Pathological examination revealed no pathological response in four patients, a major pathological response in one patient and a complete response in one patient.

**Fig. 2:**
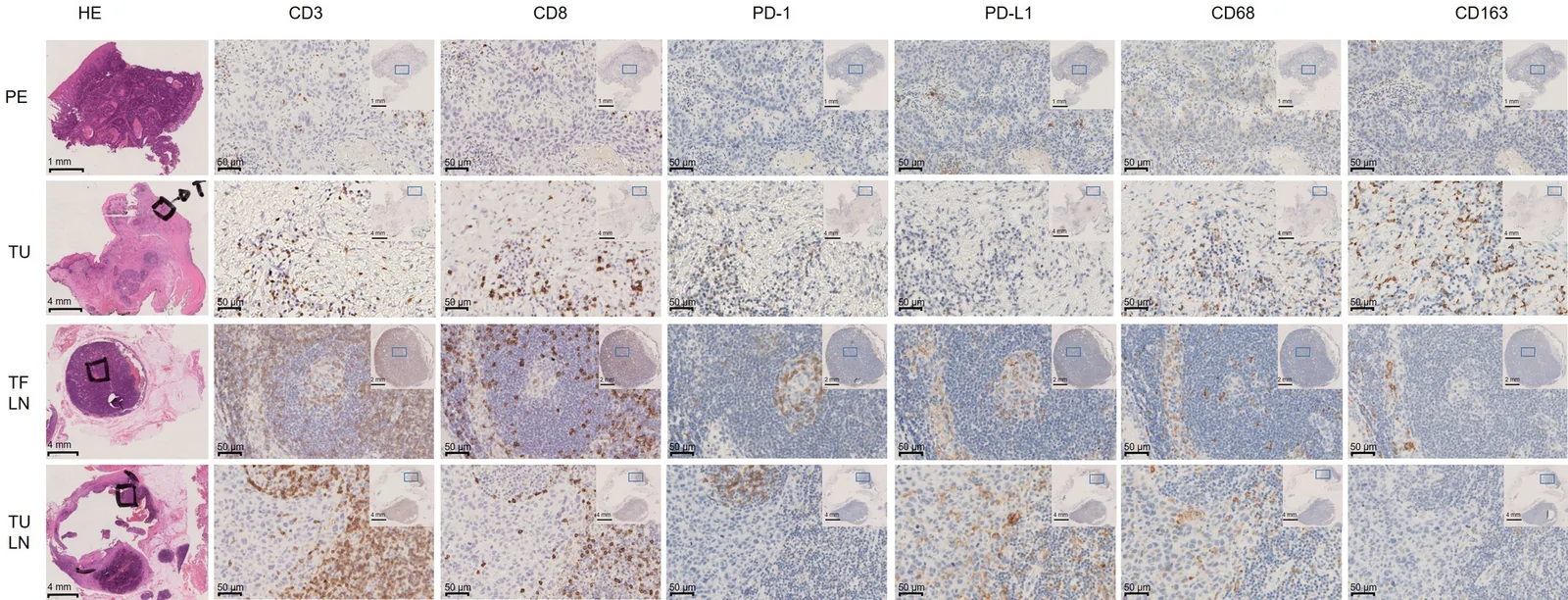
Exemplary immunohistochemical micrographs from one representative patient (patient 1; for detailed information see Table 1) at different localizations and timepoints. The figure illustrates staining characteristics and the tissue types analyzed and does not form the basis of the quantitative analysis shown in Fig. 3,the figure shows exemplary micrographs of immunohistochemical staining of one patient. The HE-Slides as well as high-power fields for CD3, CD8, PD-1, PD-L1, CD68, and CD163 are given. Tissue types of biopsy (PE), tumor resection (TU), tumor free lymph nodes (TF LN), and lymph node metastases (TU LN) are presented.

Notable differences in immune cell expression were observed between all patients before and after immunotherapy (Fig. 2). In line with the descriptive approach outlined in the Statistics section, these are reported as paired individual values per patient and compartment rather than as aggregated group comparisons.

**Fig. 3:**
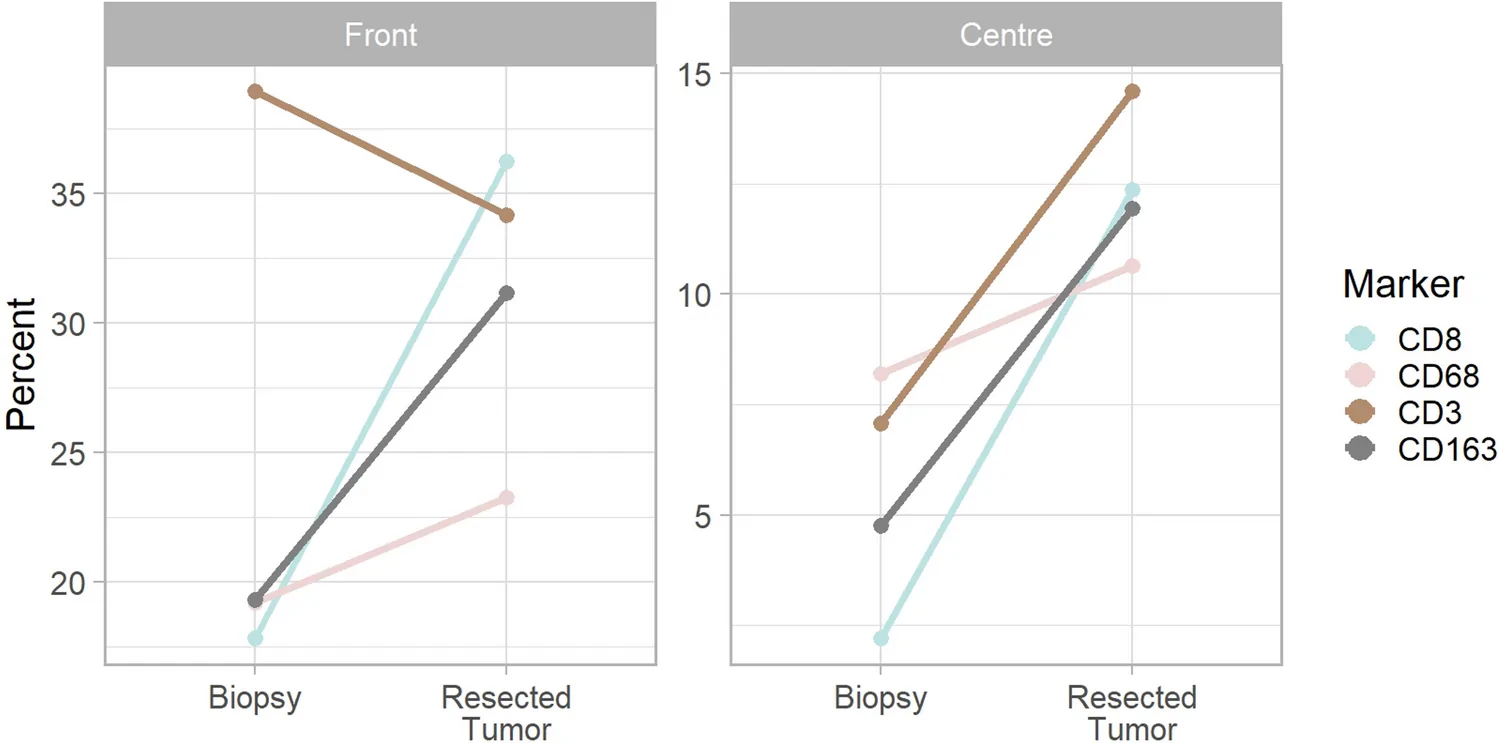
Change of immune cells before and after immunotherapy at tumor front and tumor center

At the invasive front, the most pronounced changes between the pre-treatment biopsy and the post-treatment resection specimen were observed for CD8 and CD163, with a consistent direction of change across patients, whereas the change in CD3 was small and inconsistent. At the tumor center, pronounced changes were observed for CD8, CD163 and, in contrast with the invasive front, also for CD3. Changes in CD68 were intermediate in both compartments. The paired individual values for each patient, marker and compartment, together with the direction of change, are shown in Fig. 3. As stated in the Methods, no effect sizes and no inferential statistics are reported, as the number of paired specimens does not support such analyses.

The figure shows the labeling index (positive cells/all cells) of the individual markers (CD8, CD68, CD3, CD163) in percent in biopsy and tumor resection specimens. Values for the invasive front (left) and the tumor center (right) are given.

None of the immunological markers determined in the pre-therapeutic biopsy showed a relevant correlation with response to therapy.

Regarding adjuvant treatment, two patients underwent radiotherapy, two patients underwent cisplatin-based radiochemotherapy and the patient with complete response refused further therapy after surgery.

**Fig. 4:**
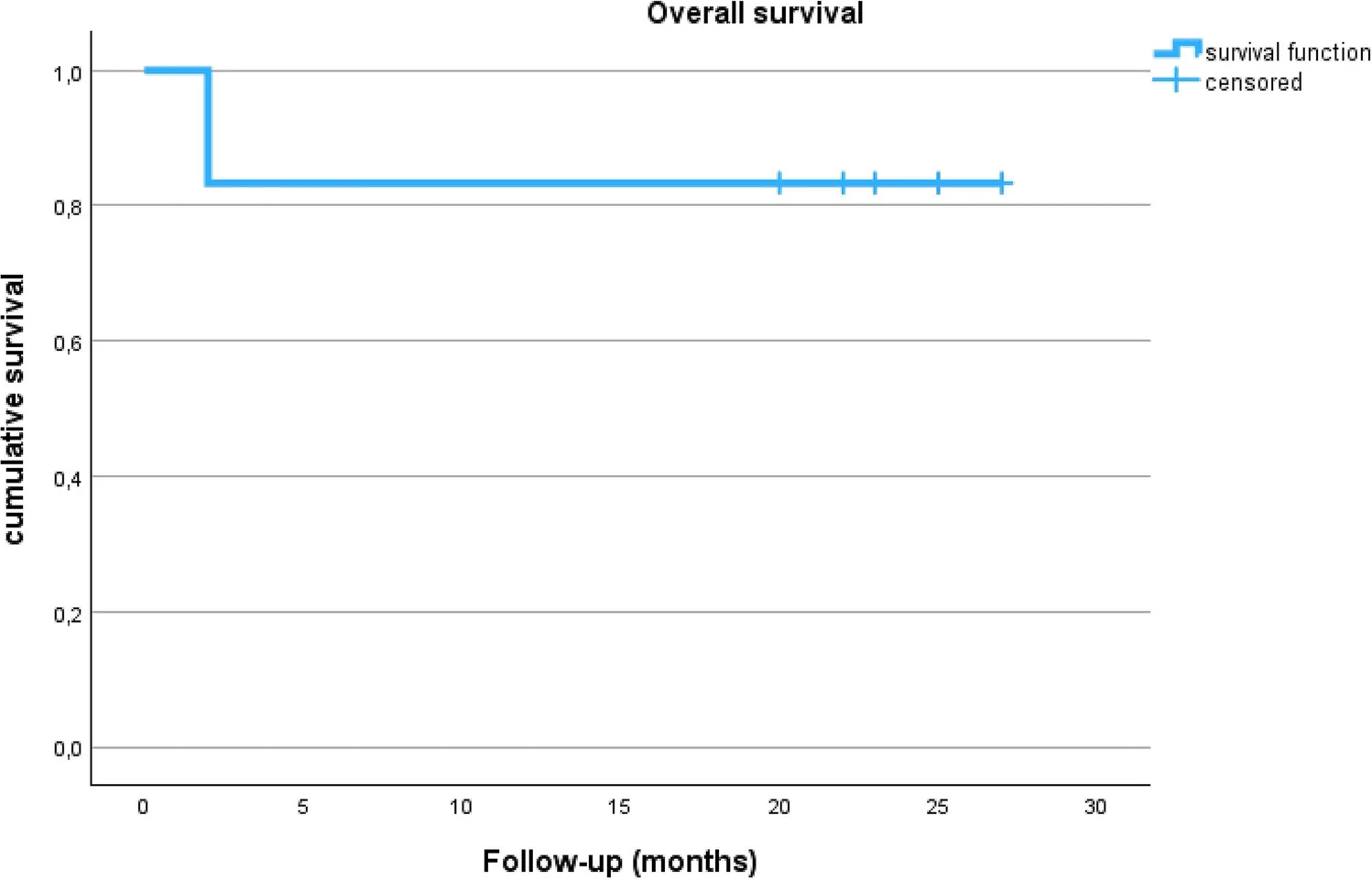
Overall survival in months

**Fig. 5:**
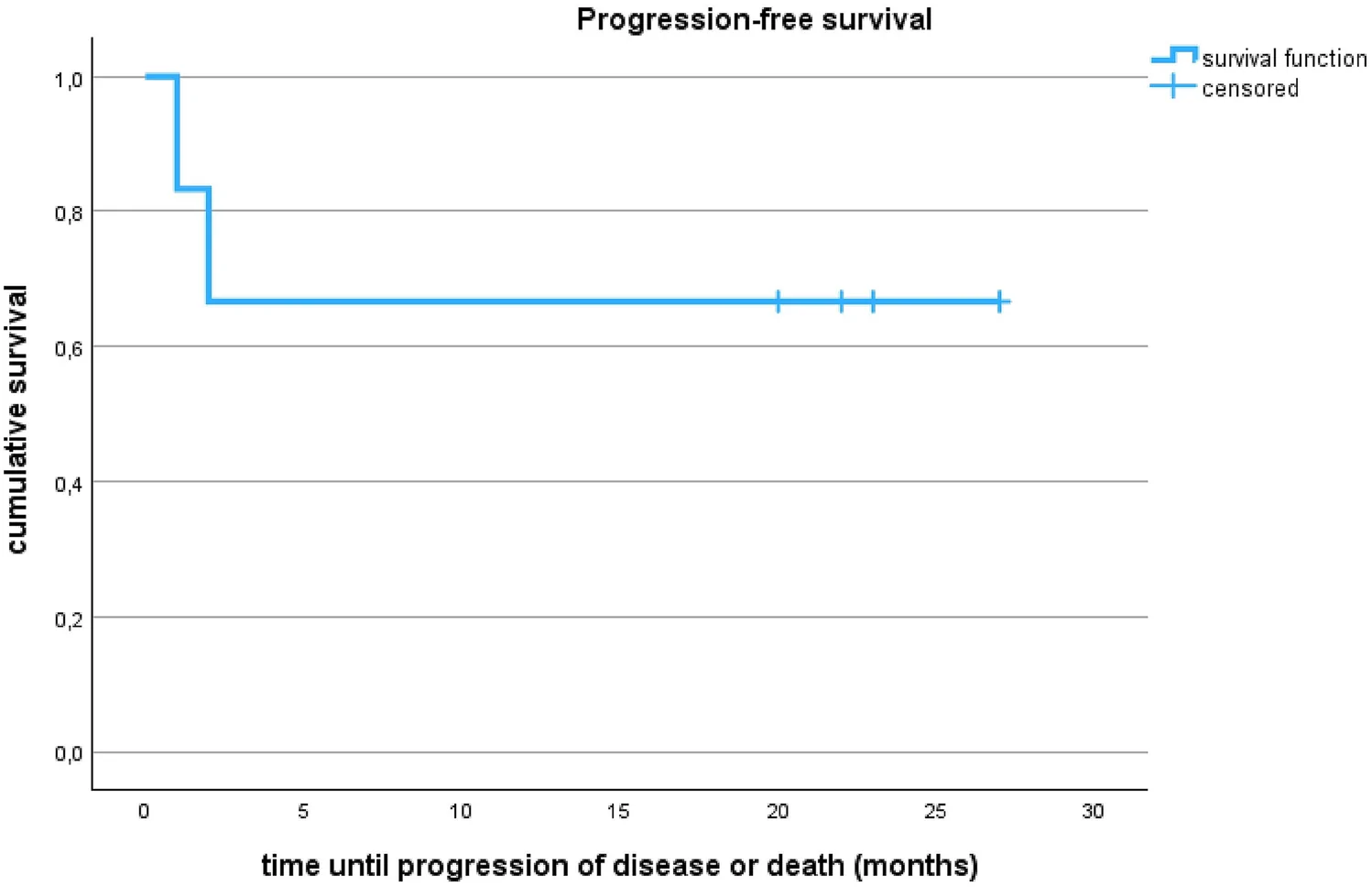
Progression-free survival in months

Two of six patients experienced a progression-free survival event within the first year: one patient showed progressive disease during neoadjuvant treatment, and one patient died postoperatively. One of six patients died during the observation period. Kaplan–Meier estimates for PFS and OS are illustrated in Figs. 4 and 5, respectively. Because of the small number of patients, these estimates are provided for illustration only and should not be interpreted as reliable survival rates.

After a median follow-up of 22.5 months, none of the patients alive and in follow-up shows recurrent disease after surgery. None of the patients being alive (*n* = 5) developed distant metastases.

**Fig. 6:**
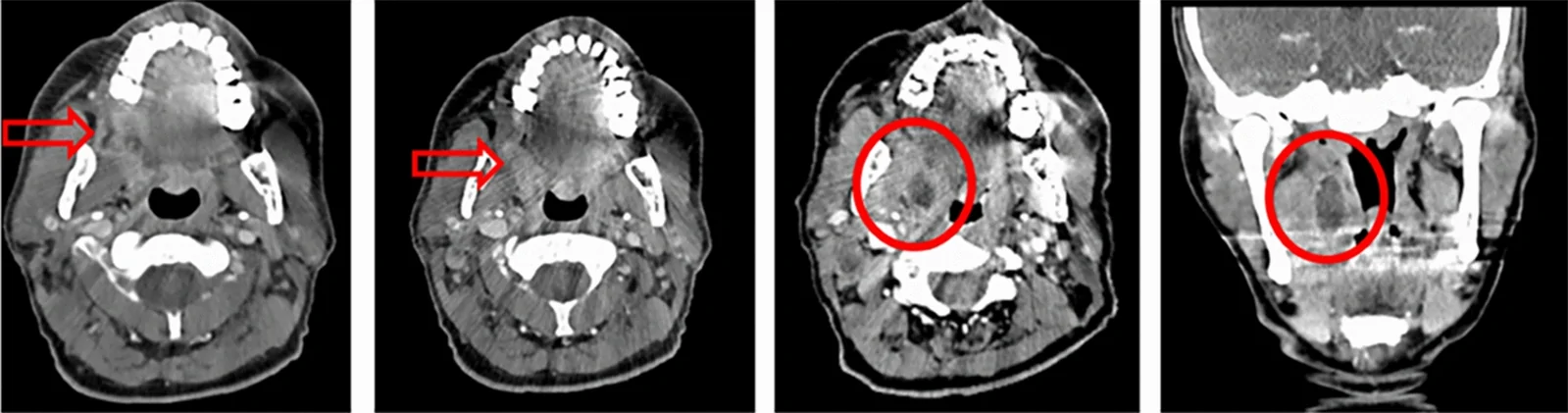
Patient 1: Radiologic signs of pseudoabscesses after TransCon TLR7/8 Agonist intratumoral injection

**Fig. 7:**
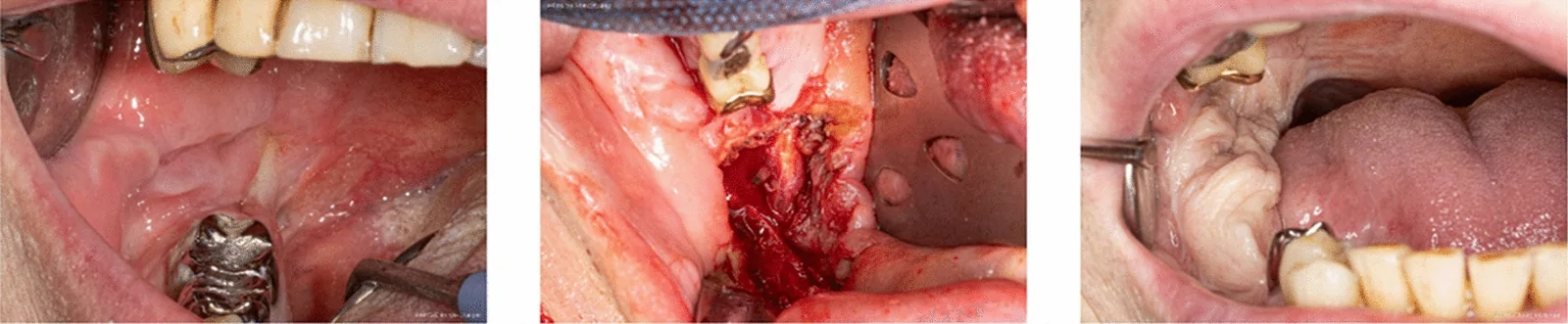
Patient 1: Clinical case presentation. The left intraoral photograph shows the patient prior to surgery, the middle photograph shows the situs during surgery after tumor resection and prior to reconstruction, the right image shows the result after surgery and microvascular reconstruction of the resection defect

The three patients with a major clinical response showed radiologic signs of pseudoabscesses after TransCon TLR7/8 Agonist intratumoral injection, see Fig. 6. However, none of these cases required additional therapy, nor did they delay or complicate surgical treatment. Figure 7 shows the retromolar tumor site of the same patient (Patient 1) before, during and after surgery.

**Fig. 8:**
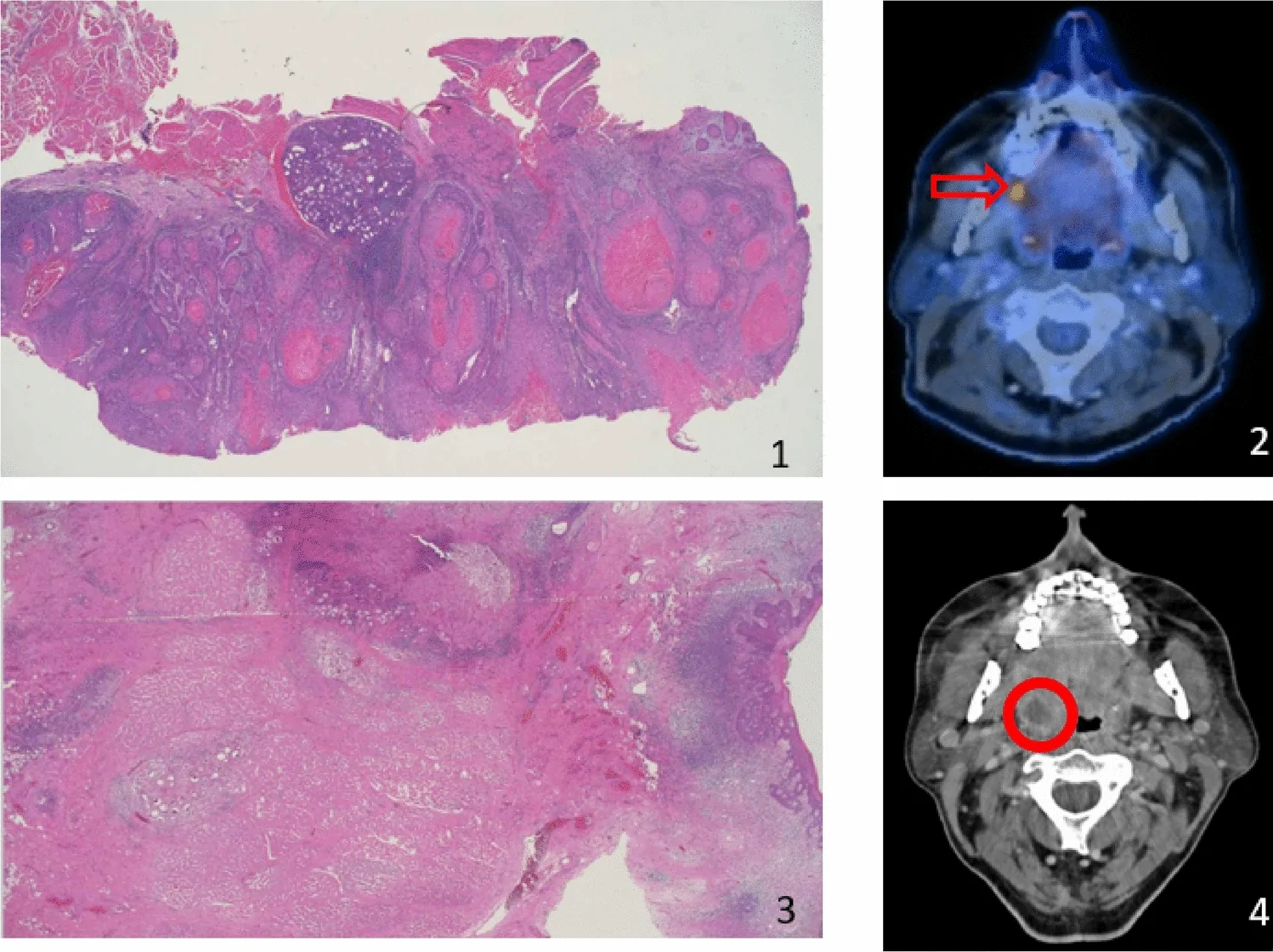
Patient 4. Histologic assessmment and radiologic imaging. Picture 1 shows HE slides (25x) of tumor biopsy of the retromolar tumor as marked in FDG-PET scan before treatment (picture 2); picture 3 shows pathologic complete response and inflammation as well as signs of pseudoabscesses as also shown in CT scan (picture 4) after second injection of TransCon TLR7/8 Agonist

The first 2 CT images show the primary tumor site prior to injection, with the tumor indicated by an arrow. Images 3 and 4 of Fig. 8 demonstrate a tonsillar pseudoabscess following intratumoral administration of the TransConTLR7/8 Agonist (Fig. 8).

## Discussion

In this single-center analysis embedded within a prospective multicenter clinical treatment study, six patients with locally advanced oral cavity squamous cell carcinoma received intratumoral TransCon TLR7/8 Agonist in combination with systemic immunotherapy using either pembrolizumab or TransCon IL-2 β/γ. Because the trial was terminated prematurely by the sponsor, the cohort size was determined externally rather than by the investigators, and all observations reported here are descriptive and hypothesis-generating.

Safety outcomes require differentiated consideration. During the neoadjuvant immunotherapy phase, all patients receiving TransCon IL-2 β/γ and one patient receiving pembrolizumab developed cytokine release syndrome (grade 1, *n* = 3; grade 2, *n* = 1); one patient developed autoimmune hepatitis leading to omission of the second cycle, and grade III events occurred in two patients. All patients proceeded to the planned surgery. The postoperative course, however, was marked by substantial morbidity: at least one grade III adverse event occurred in every patient, and one patient died postoperatively from cardiorespiratory decompensation. The treatment can therefore not be described as well tolerated without qualification. Postoperative morbidity of this magnitude is not unexpected after extensive resection with microvascular reconstruction in this patient population [18], and the events were classified as unrelated to immunotherapy by the treating physicians; given the number of patients and the absence of a control group, however, this attribution cannot be verified.

A contribution of the neoadjuvant immune modulation to the observed postoperative morbidity can indeed not be excluded. Wound healing complications occurred in four of six patients, which is at the upper end of the range expected after resection and microvascular reconstruction in this population [18]. Several mechanisms could plausibly contribute. Sustained local TLR7/8 stimulation induces a pronounced inflammatory reaction within the tissue that is subsequently resected and reconstructed, and residual inflammation at the resection margins may impair healing of the flap and of the recipient site. Systemic immune activation by IL-2 receptor β/γ agonism or PD-1 blockade may further modulate tissue repair [19], and one patient required corticosteroid treatment for autoimmune hepatitis, which itself impairs wound healing. Conversely, all patients underwent extensive multi-compartment resections with free flap reconstruction, a procedure associated with substantial morbidity irrespective of preceding systemic therapy. With six patients and no control group, these possibilities cannot be distinguished. We therefore refrain from attributing the postoperative events to any single cause, and we consider systematic prospective assessment of surgical morbidity after neoadjuvant intratumoral immunotherapy an essential component of future trials.

Clinical responses were observed in five of six patients, three of which were major responses, and occurred in the context of advanced local tumor stages at enrollment. Several patients experienced rapid symptomatic improvement, including marked pain reduction shortly after treatment initiation. Importantly, all patients remained eligible for definitive surgical resection. This observation is clinically meaningful, as neoadjuvant immunotherapy strategies raise concerns about potential disease progression that could compromise operability. In our cohort, no patient experienced treatment-related progression resulting in loss of surgical options. It should be noted, however, that clinical response was assessed by local institutional evaluation without central review and may be confounded by treatment-induced inflammatory changes, as discussed below.

Pathological assessment revealed one complete response and one major pathological response by local institutional evaluation. Although the remaining patients did not meet formal criteria for major pathological response, all resected specimens demonstrated treatment-associated changes, which is consistent with biological activity of the applied immunotherapeutic strategies but does not permit any quantification of that activity. Notably, despite all patients presenting with locally advanced disease at baseline, none of the patients currently in follow-up have developed locoregional recurrence or distant metastases after surgery and radio(chemo)therapy. Follow-up remains limited, and, as discussed below, all surviving patients received guideline-conform adjuvant treatment, so that this observation cannot be attributed to the neoadjuvant immunotherapy.

A central limitation of clinical and radiographic response assessment in this setting is the risk of pseudoresponse [20]. In our cohort, clinical and pathological response were clearly discordant. Of the three patients with a major clinical response, only two showed a major or complete pathological response, whereas one patient had a pathological non-response despite marked clinical tumor regression. Intratumoral immune activation induces edema, inflammatory infiltration, necrosis and, as observed here, sterile pseudoabscess formation, all of which may change tumor size, consistency and imaging characteristics without a corresponding reduction in viable tumor cells. Conversely, inflammatory infiltration may mask true regression. Clinical and radiographic response can therefore not be regarded as a reliable surrogate for the antitumor activity of intratumoral immunotherapy, and the pathological response of the resection specimen must be considered the more robust endpoint. Notably, the pathological response rate observed in our cohort does not support the assumption that the marked clinical responses corresponded to comparable tumor cell elimination. This discordance represents a central methodological challenge for the further clinical development of intratumoral immunotherapy in head and neck cancer and should be addressed prospectively by predefined pathological endpoints and by response criteria that explicitly account for treatment-induced inflammatory changes [21].

Sterile tumor-associated pseudoabscesses occurred in three of six patients, in two of three patients receiving TransCon IL-2 β/γ and in one of three patients receiving pembrolizumab and were observed exclusively in patients with a major clinical response. Three explanations must be considered: treatment-induced tumor necrosis with liquefaction, a sterile inflammatory reaction to the polyethylene glycol hydrogel carrier or the injected formulation, and a local immune-mediated inflammatory reaction. Our data do not permit a definitive distinction. However, two observations argue against a pure carrier or depot effect. First, all six patients received the identical formulation, carrier, dose and injection schedule, yet pseudoabscesses occurred exclusively in the three patients with a major clinical response. Second, the collections were located in the tonsillar region in spatial proximity to, but not congruent with, the injection depot, which is more consistent with a propagating inflammatory reaction than with a local reaction at the site of deposition. Local TLR7/8 stimulation induces the release of pro-inflammatory cytokines and the recruitment of myeloid cells and neutrophils, which may result in a sterile purulent-appearing collection in the absence of infection [16].

These findings should not be compared directly with large, randomized trials evaluating perioperative immunotherapy. In the KEYNOTE-689 study, neoadjuvant and adjuvant pembrolizumab demonstrated a statistically significant improvement in overall survival and progression-free survival [7]. However, pathological response rates remained relatively low, and improvements in locoregional control were modest. This discrepancy suggests that systemic immune checkpoint inhibition alone may be insufficient to induce robust local tumor regression in all patients [22, 23]. Our observations are compatible with the concept that combining systemic immunotherapy with direct intratumoral immune activation could enhance local immune responses. With six patients, the confidence interval around any response proportion spans nearly the entire range between zero and one, and the cohorts further differ in the distribution of tumor site, stage and PD-L1 expression. Random variation and selection effects can therefore not be distinguished from a true treatment effect in the present data.

None of the markers or immune cell populations centrally assessed in the preoperative biopsy showed a meaningful correlation with clinical and/or pathological tumor response. However, this finding is very likely limited by the small sample size. Our own immunohistochemical analyses of biopsy and tumor resection specimens revealed an increase in myeloid and T-cell infiltration especially in the tumor center, which is the compartment adjacent to the injection depot and therefore the region in which a local effect would be expected. The intratumoral administration of a TLR7/8 agonist may directly modulate the tumor microenvironment by activating innate immune pathways, promoting antigen presentation, and enhancing T-cell infiltration [24]. Such mechanisms could synergize with systemic immune checkpoint blockade or cytokine therapy, thereby amplifying antitumor immunity both locally and systemically. We emphasize that this remains a hypothesis that is consistent with, but not demonstrated by, the present observations. On the existing haematoxylin–eosin and immunohistochemically stained whole-slide sections, the tissue adjacent to the pseudoabscess showed a foreign body reaction with abundant giant cells, epithelioid cells, a mixed and lymphocyte-dominant inflammatory infiltrate and thickened secretions. Centrally, within the pseudoabscess, necrosis with cellular debris is evident. Nevertheless, based on our clinical experience, further prospective trials evaluating combined intratumoral TLR activation and systemic immunotherapy strategies appear highly warranted.

This study has several important limitations that must be acknowledged. First, the number of included patients is small, and two different systemic treatment regimens were applied, which introduces heterogeneity and limits direct comparability. Second, the single-center design restricts generalizability. Third, clinical and radiographic responses were assessed by local institutional evaluation without central review and are subject to the risk of pseudoresponse discussed above. Fourth, the translational analyses were performed on paired specimens from six patients only, and no formal spatial profiling of the injection region was possible. Fifth, and importantly for the interpretation of the survival outcomes, all surviving patients received guideline-conform postoperative treatment: two patients underwent radiotherapy, two patients received cisplatin-based radiochemotherapy, and one patient declined further treatment after surgery. Adjuvant radio(chemo)therapy is itself an established determinant of locoregional control and survival in locally advanced oral squamous cell carcinoma [25], so that the absence of locoregional recurrence in the patients currently in follow-up cannot be attributed to the neoadjuvant immunotherapy. Consequently, no inferential statistics were performed, and the results should be regarded as hypothesis-generating rather than definitive.

In conclusion, this monocentric analysis of a prematurely terminated prospective randomized phase 2 trial shows that neoadjuvant intratumoral immune stimulation combined with systemic immunotherapy is feasible in patients with locally advanced oral cancer, that all patients remained eligible for definitive surgery, and that the treatment was accompanied by consistent remodeling of the tumor immune microenvironment. At the same time, pathological responses were heterogeneous and discordant from clinical responses, and postoperative morbidity was substantial. No conclusion regarding efficacy can be drawn from six patients. The observations reported here, in particular the sterile tumor-associated pseudoabscesses and their association with major clinical response, are hypothesis-generating and define concrete questions—predefined pathological endpoints, response criteria accounting for inflammatory change, prospective sampling of the injection region, and systematic assessment of surgical morbidity—for future prospective trials of combined intratumoral and systemic immunotherapy.

## Data Availability

The data supporting the findings of this single-center analysis are available from the corresponding author upon reasonable request. Data from the parent trial are held by the sponsor.
